# Indirect Confirmation of a COVID-19 Encephalitis Case

**DOI:** 10.7759/cureus.36959

**Published:** 2023-03-31

**Authors:** Kleoniki Georgousi, Panagiotis Karageorgiou, Maria Tzaki, Ioanna Pachi, Ioannis Kyriazis

**Affiliations:** 1 Internal Medicine/Infectious Diseases, KAT Attica General Hospital, Kifissia, GRC; 2 Internal Medicine, KAT Attica General Hospital, Kifissia, GRC; 3 Neurology, National and Kapodistrian University of Athens, 1st Neurology Clinic, Eginition Hospital, Athens, GRC; 4 Internal Medicine/Diabetes, KAT Attica General Hospital, Kifissia, GRC

**Keywords:** encephalitis, cerebrospinal fluid, central nervous system, sars-cov-2, covid-19

## Abstract

A 58-year-old man was admitted to the hospital with acute neurological manifestations of encephalitis 15 days after a previous upper respiratory COVID-19 illness. On presentation, he was confused with altered mental status, aggressive behavior, and a Glasgow coma scale score of 10/15. Laboratory investigation, brain computed tomography (CT), and brain magnetic resonance imaging (MRI) were unremarkable with normal results. Although the cerebrospinal fluid (CSF) polymerase chain reaction (PCR) for SARS-CoV-2 was negative, we found increased concentrations of positive immunoglobulin (Ig) A and IgG antibodies in CSF, suggesting acute central nervous system (CNS) infection and indirect confirmation of virus neuroinvasion. There was no evidence of humoral auto-reactivity, and we rejected the hypothesis of autoimmune encephalitis with known autoantibodies. On the fifth day of hospitalization, myoclonic jerks emerged as a new neurological sign until the added levetiracetam led to total remission. The patient achieved full recovery after antiviral and corticosteroid therapy implementation of 10 days in the hospital. This case report emphasizes the importance of the presence of CSF IgA and IgG antibodies to diagnose encephalitis in COVID-19 patients as an indirect confirmation of CNS infection.

## Introduction

Although neurological manifestations in COVID-19 illness are seen in one-third of cases, diagnosing encephalitis is a challenging issue due to the scarcity of the findings on cerebrospinal fluid (CSF) analyses [1]. Besides the clinical evidence of neurological involvement, in most cases, the polymerase chain reaction (PCR) for SARS-CoV-2 is negative in the CSF [2,3], whereas the exception of this rule with positivity of virus CSF PCR is supported by only a few reported cases. There are even fewer reports of encephalitis confirmed by the presence of SARS-CoV-2 antibodies in CSF [4-6]. We present a case of indirect confirmation of COVID-19 central nervous system (CNS) infection by SARS-CoV-2 antibodies detection in CSF.

## Case presentation

A 58-year-old man was admitted to the emergency department due to abrupt onset of altered mental status and acute behavioral changes in March 2022. His medical history was significant for hypertension and hypothyroidism under medication and complete vaccination for SARS-CoV-2 with three doses of mRNA vaccine. According to his relatives, he was diagnosed with mild COVID-19 due to upper respiratory symptoms and positive rapid antigen test for SARS-CoV-2 15 days before presentation.

At presentation, he had a mild fever of approximately 37.5°C, diaphoresis, palpitation, and oxygen saturation at 98% in room air, without clinical signs of respiratory infection. On neurological examination, the patient was confused with increased agitation, aggressive behavior, and disorientation in time, space, and person. He had language disturbances and was unable to have a simple conversation. The Glasgow coma scale score at this time was nearly 10/15; however, he was quadrikinetic with normal papillary reflex and isocoria, no cervical rigidity, and no lateralization in cranial and motor nerve examination. The potential sensory disturbances could not have been evaluated at that time.

Reverse transcription PCR (RT-PCR) assay for SARS-CoV-2 using a nasopharyngeal swab showed positive results, whereas the rapid antigen test for SARS-CoV-2 was negative. The complete blood count was unremarkable with a white blood cell count of 10,400 cells/μL (4,600-10,200 cells/μL), neutrophils of 2,990 cells/μL, lymphocytes of 2,440 cells/μL, hematocrit of 44.9%, hemoglobin of 14.9 g/dL, platelet count of 233,000, C-reactive protein of <0.3 mg/dL, and procalcitonin of 0.02 ng/mL, except from increased D-dimer level of 0.8 mg/L (<0.5).

Biochemical analyses showed elevated lactate dehydrogenase of 392 IU/L (normal range: 125-243), elevated creatine phosphokinase of 371 IU/L (normal range: 30-200), and ferritin of 334 ng/mL (normal range: 21.8-274.7), whereas serum virological testing of hepatitis B virus, hepatitis C virus, human immunodeficiency virus, and syphilis were negative. Chest X-ray was unremarkable, followed by computed tomography pulmonary angiogram (CTPA), which ruled out pulmonary embolism or other pathological findings in the lungs. Brain CT revealed a normal image with no ischemic or hemorrhagic lesions. A lumbar puncture took place, and CSF analyses showed that the CSF was clear with mild pleocytosis, leukocyte count of 12/μL (unable to differentiate), glucose of 67 mg/dL, a slight increase of protein of 46.5 mg/dL (normal range: 15-45 mg/dL), and lactate dehydrogenase of 13 IU/L, all within the normal limits.

CSF examination for bacteria, viruses, and yeast via FilmArray assay and culture was negative. CSF real-time PCR for SARS-CoV-2 was negative. However, the concentrations of anti-spike (anti-s1) antibodies immunoglobulin (Ig) A (6.31; positive ≥1.1) and IgG (3.33; positive ≥1.1) with enzyme-linked immunosorbent (ELISA) assay exceeded the upper reference levels in the CSF. The notably increased IgA levels confirmed acute infection through lack of IgM testing due to laboratory deficiencies.

Other immune-mediated neurological disorders, such as autoimmune encephalitis, stiff-person syndrome, progressive encephalomyelitis with rigidity, and myoclonus (PERM) syndrome were excluded via autoantibodies detection with indirect fluorescent antibody (IFA) assay in CSF. Specifically, IgG autoantibodies to 65-Kda glutamic acid decarboxylase, glycine receptor (GlyR), and neuronal cell-surface antigens in the central nervous system (AMPA-R1/2, GABAB-R, LGI1, DPPX, GluRδ2, mGluR5, CASPR2) were detected as negative. IgG N-methyl-D-aspartate (NMDA) receptor autoantibody was not feasible to be measured due to laboratory incapacity (Table 1).

**Table 1:** Cerebrospinal fluid analyses ELISA, enzyme-linked immunosorbent; HH6, human herpesvirus 6; HSV, herpes simplex virus; IFA, indirect fluorescent antibody; Ig, immunoglobulin; LDH, lactate dehydrogenase; RT-PCR, reverse transcription polymerase chain reaction; VZV, varicella zoster virus

Cerebrospinal fluid	Values	Reference range
Color	Colorless	Colorless
White blood cell count (mm^3^)	12	0-5
Red blood cells (mm^3^)	0	0
Glucose (mg/dL)	67	40-70
Protein (mg/dL)	46.5	15-45
LDH (IU/L)	13	< 20.0
Escherichia coli PCR	Negative	Negative
Haemophilus influenzae PCR	Negative	Negative
Listeria monocytogenes PCR	Negative	Negative
Neisseria meningitidis PCR	Negative	Negative
Streptococcus agalactiae PCR	Νegative	Negative
Streptococcus pneumoniae PCR	Negative	Negative
Cytomegalovirus PCR	Negative	Negative
Enterovirus PCR	Negative	Negative
HSV1-2 PCR	Negative	Negative
HH6 PCR	Negative	Negative
Human parechovirus PCR	Negative	Negative
VZV PCR	Negative	Negative
Cryptococcus neoformans/gatti PCR	Negative	Negative
Culture	Negative	Negative
SARS-CoV-2 RT-PCR	Negative	Negative
SARS-CoV-2 IgA (ELISA)	6.31	Negative: <0.8; Doubtful: 0.8-1.09; Positive: ≥1.1
SARS-CoV-2 IgG (ELISA)	3.33	Negative: <0.8; Doubtful: 0.8-1.09; Positive: ≥1.1
Anti-glutamic acid decarboxylase IgG (IFA)	<10	<10
Anti-glycine receptor/GlyR IgG	Negative	Negative
Anti-AMPA-R1/2 IgG	Negative	Negative
Anti-GABAB-R IgG	Negative	Negative
Anti-LGI1 IgG	Negative	Negative
Anti-DPPX IgG	Negative	Negative
Anti-GluRδ2 IgG	Negative	Negative
Anti-mGluR5 IgG	Negative	Negative
Anti-CASPR2 IgG	Negative	Negative

The patient was admitted to a COVID-19 clinic, and intravenous mannitol, haloperidol, and isotonic fluids along with subcutaneous enoxaparine sodium were started. Dexamethazone 24 mg per day (days 1-5) tapered to 12 mg per day (days 6-10) and remdesivir 200 mg once daily on the first day and 100 mg once daily from the second day were added to the therapeutic scheme. There was no need for oxygen administration.

Dramatic neurological improvement was achieved in the next two days, and brain MRI with and without contrast was performed, which revealed normal results with no pathological contrast enhancement. On the fifth day of hospitalization, the patient had already regained full consciousness and was alert physically and mentally. On repeated neurological examination, he had a Glasgow coma scale score of 15/15 and was well oriented in time, space, and person with good speech apart from newly emerged myoclonic jerks of the lower limbs. Intravenous levetiracetam 500 mg twice a day was added to the previous therapy, as deemed necessary. After 10 in-hospital days, our patient had achieved a full recovery and complete resolution of neurological symptoms and signs, and he was discharged home with the only recommendation for levetiracetam continuing at a tapering dose, which finally stopped after a two-month follow-up.

## Discussion

SARS-CoV-2, through its spike protein interaction with ACE2 (angiotensin-converting enzyme 2) receptor, enters the blood stream and spreads to systems and organs, including the central and peripheral nervous systems. In various epidemiological studies, the frequency of neurological symptoms in COVID-19 patients can reach 30%, emphasizing the potential neurotropic character of SARS-CoV-2 [7-10].

Nervous system infection occurs via two routes: the hematogenous route, where the virus infects the endothelial cells of the brain directly and crosses the blood-brain barrier, and the neuronal route, where the virus spreads by retrograde-anterograde transport from the olfactory, respiratory, and enteric nerve ends to the brain [11]. Neurological manifestations are divided into a) central nervous system manifestations such as headache, dizziness, meningitis, encephalitis, acute disseminated encephalomyelitis, autoimmune encephalitis associated with known autoantibodies, tranverse myelitis, stroke, and seizures, b) peripheral nervous system manifestations, which include anosmia, ageusia, Guillain-Barre syndrome, and its variant Miller Fisher syndrome, and c) skeletal muscle manifestations [11].

In COVID-19 patients, the occurrence of encephalitis is not a typical phenomenon and can present before, parallel to, or after respiratory manifestations of the illness. The latency between the respiratory illness and encephalitis manifestations ranges from over two weeks to 40 days [12]. In our case, the patient had upper respiratory symptoms 15 days before admission. We assume, for that reason, that CNS infection took place by the neuronal route via the retrograde-anterograde transport of the virus from the olfactory nerve ends to the brain, upon the nasal infection.

Based on a systematic review of 79 studies [13], encephalitis patients were 52.7% male and 85.6% adults (49.3 ± 20.2 years). In another review of case reports and case series [14], the frequency of neurological manifestations was as follows: altered mental status in 53.7% of the cases, decreased consciousness or unconsciousness in 33.3%, seizure in 29.62%, general symptoms such as fever in 70.37%, headache in 20.37%, and myoclonus in 5.55%. In the same study, the most common comorbidities of the affected patients were hypertension (29.16%), diabetes mellitus (14.58%), and, less commonly, hypothyroidism (2.08%). All of the above demographics and clinical parameters were matched with our patient’s relative characteristics in the setting of encephalitis.

The most common findings on CT scan images are hypodensities in white matter (17.14%) and cerebral hemorrhages/hemorrhagic foci (11.42%). The most common findings on MRI are hyperintensities in white matter (44.68%), the temporal lobe (17.02%), and the thalamus (12.76%). In our case, brain CT/MRI was normal possibly due to either early investigation for brain changes soon after the onset of neurological symptoms or the patient having a milder form of encephalitis. Moreover, it has been postulated that MRI changes can sometimes be transitory, and the only way they can be shown is via positron emission tomography (PET) imaging [15,16].

The first reported cases of encephalitis were reported in China in early March 2020 and in Japan in April 2020 [10,17,18]. In most encephalitis cases, only a small proportion have reported CSF PCR positive for SARS-CoV-2. In addition, a subset of COVID-19 patients with neurological disturbances have CSF immune responses to both direct virus invasion in CNS and anti-neural autoimmunity. In a previous systematic review [4], only one case was reported by the presence of SARS-CoV-2 antibodies in CSF. In another recent systematic review [5], there was only one case with positive CSF IgG and IgM antibodies for SARS-CoV-2 in a patient who presented with acute inability to walk, coprolalia, and persecution delusion. In the same study, there was also one case with positive PCR and IgG antibodies for SARS-CoV-2 and additional detection of anti-NMDA antibodies in CSF in a man with altered behavior and mental status.

In an exploratory immune CSF analysis of patients with acute COVID-19 and neurological symptoms, Song et al. revealed a compartmentalized immune response to SARS-CoV-2 different from that in the periphery, involving innate and adaptive immunity [6]. They revealed increased interleukin (IL)-12 and IL-1b levels in CSF, but not in plasma, and B cell responses to SARS-CoV-2 by the presence of CSF anti-s1 and anti-nucleocapsid antibodies, with a different profile from those in plasma in the setting of CNS infection.

In the same study, the authors found a cross-reactivity of some CSF-expanded B cell populations against self-antigens with an elevated burden of intrathecal autoantibodies, including anti-neural antibodies. PCR for SARS-CoV-2 in the CSF was negative in all participants. The authors suggested that the negative PCR assay for SARS-CoV-2 in the CSF of patients with neurological symptoms may mean either the presence of low and undetectable CSF viral load, leading to false negative results, or the transient passage of the virus in the CNS, leaving behind indirect proof of invasion with the presence of antibodies [19].

In our case, the patient had acute neurological manifestations of encephalitis with mild pleocytosis and a slightly elevated protein level in the CSF. Although the CSF PCR for SARS-CoV-2 was negative, we revealed increased concentrations of positive IgA and IgG anti-s1 antibodies, suggesting indirect confirmation of CNS infection. The presence of CSF anti-s1 antibodies means either recent vaccination or acute infection, with the former explanation being invalid in our case because the patient had his last shot of vaccine eight months before admission. The investigation of anti-neural antibodies in CSF in the context of a broader CNS humoral autoimmunity was negative, and the hypothesis of autoimmune encephalitis with known antibodies was abandoned.

Based on the literature, the management of COVID-19-related encephalitis consists of intravenous methylprednisolone, oral prednisone, intravenous immunoglobulin, and plasma exchange. By these treatment options, there has been clinical improvement in 37.8%, full recovery in 28.9%, and lethal outcomes in 20% of encephalitis patients [13]. In our case, given the acute COVID-19 illness with neurological impairment even after the 15-day latency from upper respiratory symptoms, we decided on the implementation of antiviral and corticosteroids therapy, which ended with a successful result.

## Conclusions

In spite of the usual negative results in the PCR for SARS-CoV-2 in the CSF of COVID-19 patients who present with acute neurological manifestations, there are some noteworthy findings in the CSF analyses that could lead to encephalitis diagnosis in an indirect manner. We highlight the importance of the presence of CSF SARS-CoV-2 IgA and IgG antibodies by means of virus neuroinvasion and CNS infection, causing COVID-19 encephalitis. In addition, it is important in patients with neurological impairment to exclude the possibility of autoimmune encephalitis with known antibodies.
